# Fulton County Medical Society

**Published:** 1871-03

**Authors:** Charles Pinckney


THE GEORGIA

### MEDICAL COMPANION:

A MONTHLY ADVISER.

VOL. I. ATLANTA, GA., MARCH, 1871. NO. 3.

### PART I.

### Original Communications and Special Selections.

### FULTON COUNTY MEDICAL SOCIETY, ATLANTA, GA.

*Extracts from the Minutes of February* 20, 1871. *Reported by* CHARLES PINCKNEY, A. M., M. D.

*Dr. T. S. Powell* reported two cases of cerebro-spinal Meningitis. The first was a white man, aged 19, of sanguine temperament and worked at a butcher stall. On the 29th of January, 1871, he complained of indisposition, quit work, took a few drinks to dissipate his bad feelings, and went home to bed. Next morning found him drowsy, with nausea and acute pain in the head and spine. He vomited freely. In the evening I was sent for, and discovered the patient in a semi-comatose condition. When aroused, he complained of the symptoms above enumerated, the pulse being feeble and slow, the skin clammy and eyes congested. There was some contraction of the posterior muscles of the neck, and the medulla oblongata was no doubt seriously involved. The patient, as indicated by the pulse and skin, was already on the verge of dissolution. I prescribed hydrate of choral and bromide of potassium, together with counter-irritants to the spinal column. The disease, however, had progressed too far to admit of any hope of arresting it. The treatment palliated, but failed to control, and he died about 34 hours after my first visit.

The second case was a negro carpenter, aged 28, robust, vigorous and healthy, but dissipated and irregular in his habits. On the night of the 3d of January, 1871, he went to bed as well as usual, save that his bowels were constipated. Next morning, he complained of chilly sensations, headache and a "crick in the neck;" pain along the spinal column, soreness of the extremities and muscles generally. In the afternoon I was called in and found him delirious, with a hot and dry skin, pulse frequent and hard, tongue coated white, bowels bound and eyes congested. He said that his head hurt him "all over" and that his limbs were very sore and stiff. His head was drawn backward, which readily accounted for the "crick in the neck." I ordered a mustard plaster applied to the entire length of the spine, a hot mustard foot bath, cold water, in liberal quantity, to the head, and 15 grs. of Calomel. Six hours after, the mercurial had acted twice largely. The pulse had softened, and was less frequent, the skin not so warm, and the patient more quiet; but there was delirium still, and some fever. Deeming the remission sufficiently marked to warrant the administration of quinine, I gave 10 grs. of that drug, and ordered five more to be given in two hours. Next morning he was better, as indicated by an improved condition of the pulse and skin. Delirium still persisted to some extent. Towards midday, he had an accession of fever, with increased restlessness and delirium. I ordered the hot mustard foot-bath repeated, and cold water to the head as before. By night, the fever began to decline, and there was a corresponding abatement of delirium. I prescribed:

℞—Quiniæ Sulph. . . . grs. xv.
Ft. Chart No. 3.

S. One powder every two hours during the night. The first dose was given at 10 o'clock, P. M. The following morning found my patient with skin and pulse much improved and but little fever. He, however, still complained of soreness about the neck and extremities, and gave occasional evidences of delirium. Mustard pediluvium and spinal counter-irritation were repeated, omittiug quinine at night. The next day his pulse indicated some febrile reaction, in view of which the quinine was resumed at night as before. On the following day he still complained of his neck and general soreness, and there was strabismus of one eye, but he had no fever. The patient was then put on iodide of potassium, in doses of four grains every six hours, under which he improved rapidly and was soon restored to health.

*Dr. E. J. Roach* remarked that his experience in the treatment of Meningitis induced him to believe that the stimulating method afforded the best prospect of success. While in charge of Floyd House Hospital, at Macon, Georgia, during the late war, the disease at one time assailed quite a number of patients under his care. On its first appearance, his assistants reported them as very unique cases. The patients would complain of debility and excessive prostration, followed by rigors, which soon resulted in complete opisthotonos. The first cases terminated fatally, and so early after the attack that very little, if any, treatment was instituted. Adopting the stimulating theory, his assistants were requested to administer large doses of brandy, on the first appearance of prostration or of the chill, and the result was that many recovered. Whenever a patient passed through the chill into complete opisthotonos, death invariably ensued.

*Dr. Powell:* I would ask, Doctor, what seemed, in your opinion, to be the cause of the cases which occurred under your observation?

*Dr. Roach*: Of that I formed no decided opinion. The patients were all from lower Georgia, and had been subjected to malarial influence. Miasm may have been the predisposing cause. I observed that the disease was confined to one command, and that all of those attacked with cerebro-spinal meningitis had previously suffered from intermittent fever.

*Dr. Powell:* While stimulants might in many instances, eliminate the peculiar poison producing the disease, yet I think it probable that quinine would have accomplished much in the class of cases mentioned by Dr. Roach. It should be given without regard to the condition of the brain, at the first remission of fever, and be repeated every day, at the same time, for several days.

*Dr. W. T. Goldsmith* said he did not believe in specific medication. Age, sex, temperament and peculiarities of constitution would impress various forms of the same disease upon different persons. The asthenic form of this malady, which was the usual one in sporadic cases in the adult, demanded often the employment of the lancet. In such he had used it with great benefit. Here, too, the iodide of potassium, and mercury, given in large doses, frequently repeated, are remedies worthy of the highest consideration. He would have the patient stripped and enveloped in blankets wrung out in hot water, and repeat the operation as often as necessary, after which counter-irritants should be applied to the spinal column. As auxilliary to the treatment, chloral hydrate would be useful in allaying excitement and quieting the nervous system. Sometimes, meningitis, from the start, assumed an asthenic character. In such cases, stimulants, opium, chloral would be indicated, and quinine, of course, should marked remission occur at any time in the fever. He believed that each case, in a measure, demanded a mode of treatment peculiar to itself. The secondary fever required no less care and attention, as it often proved fatal after the patient had passed safely through the alarming ordeal of the primary stage. As to the cause, he believed meningitis to be zymotic in its origin—a specific manifestation of blood-poisoning, closely allied to that of puerperal fever and erysipelas, assailing the membranes of a vital organ. While not an exanthematous disease, yet it frequently assumed that form, in an abortive effort, perhaps, to eliminate the poison from the blood. In this form it was originally called "spotted fever."

*Dr. Powell:* Have you ever seen or heard of a case in which the disease assumed the spotted form in this country ?

*Dr. Goldsmith:* I have not ; neither have I ever seen a case of typhoid fever, with marked *petechiæ*, in this country, yet it is one of the characteristics of the disease in other parts of the world.

*Dr. C. Pinckney*: Petechiæ, in typhoid fever, was of common occurrence on the coast of South Carolina in the years 1856, 1857 and 1858. I have often seen it in that locality. In one instance, these spots, instead of subsiding, degenerated into ulcers about the neck, chest, back and extremities, some of which were deep and large enough to admit the end of the finger. The patient, an interesting young lady, after months of torture, recovered perfectly. Two other cases, of precisely similar character, occurred about the same time, in the same family, both of which terminated fatally.

During the winter of 1869, I treated fourteen clearly-marked cases of meningitis, one half of which were diagnosed to be cerebro-spinal. Eight of these cases recovered. The phenomena presented by some of them were very like a paroxysm of pernicious intermittent fever; in the others, the development was gradual. In all, cephalagia, generally frontal, was a prominent symptom. I am inclined to doubt whether meningitis, as observed in this particular locality, can be termed a distinct idiopathic affection. Like the bronchitis of influenza, or *la grippe* of the French, it is probably but a local manifestation of a constitutional malady—a symptom incidental to the general disease. In 1869, to which my experience of meningitis is confined, its prevalence was synchronous with epidemic bronchitis, and there is reason to believe that both affections were produced by the same special atmospheric cause. When it attacked the bronchiæ, it was called influenza; when it inflamed the membranes of the brain and spinal marrow, it was called cerebro-spinal meningitis. The meningitis of our section is perhaps identical with the epidemics of Great Britain and the continent, and the spotted fever of New England in the beginning of the present century, modified and mollified, however, by local circumstances. The type known to us is less malignant in its character—less fatal. However, epidemics, in Atlanta, have never, to my knowledge, assumed a very malignant form. I believe that this affection results from a toxical condition of the blood, and that each case requires its own peculiar treatment. What might cure one, would kill another. The therapeutic means used to combat meningitis, are various—sometimes antagonistic, and even paradoxical.* Some bleed where stimulants are necessary. My plan is to study each individual case, and strive to meet the indications peculiar to it. At the same time, I feel

*Mercury, in doses large and small, purgatives, counter-irritants, tartar, ice, chloral, gelseminum, quinine—remedies without limit have been brought to bear upon this formidable disease.

justified in doing or giving anything which seems to offer a reasonable prospect of success.

*Dr. Powell:* I agree, to some extent, with Drs. Goldsmith and Pinckney, that the disease is the result of a blood-poison, akin to that of erysipelas in its effects upon the system; and that when it prevails epidemically, it does, in many respects, bear a very close resemblance to influenza. I have never seen it epidemic, but have met with quite a number of sporadic cases. In a majority of them I think I was able to trace the cause to exposure, and to bad or unwholesome diet. Though unprepared, just now, to offer an argument satisfactory to myself as to the influence of bad diet upon the brain and nervous system, yet it is clear that meningitis was not so prevalent in the good old days of "peace and plenty"—when everybody made his own meat and smoked it—when the corn was carefully selected and "nubbed" before being turned into meal—when fresh vegetables grew in every garden, and the orchards and vineyards teemed with fruit. Now, these articles, after being subjected to fermentation, and in a state of semi-decay, are largely imported by wanton speculators for purposes of home consumption. It it a well-known fact that damaged corn will, in horses, produce "blind staggers" as a poison acting ultimately upon the brain of the animal. According to my observation, there have been more cases of nausea, vomiting and vertigo since the war than during the preceding twenty years, in the same community, which is due, I have no doubt, in a great measure, to bad diet—imported food. A scientific inspector of food for our cities, is far more important, in a hygienic point of view, than any board of health. The day is not far distant, I trust, when the one will supersede the other.

*Dr. Pinckney:* There may be much truth in the remarks of Dr. Powell, yet there is hardly sufficient data to warrant his wide conclusions as to the influence of bad diet in the production of the disease under consideration. Blind staggers, in horses, is a kind of apoplexy, which may depend upon a variety of causes, such as indigestion, constipation, heat, or excessive exercise. It not unfrequently occurs without reference to the character of ingesta. Now, it will be observed, in the two cases reported by my friend, Dr. Powell, that the first worked at a butcher-stall. It is presumable that he had always on hand a full supply of sound, wholesome meat, and therefore lived well. Yet that man was attacked with meningitis, and died of it in a few hours. The second case occurred in the person of a negro carpenter, of dissipated habits. The presumption is that he drank a great deal of mean whiskey—the vilest form of corn. That man experienced a severe attack of meningitis, and notwithstanding his irregular habits, recovered. While bad diet is certainly a predisposing cause of disease, I am not prepared to believe that it is peculiarly inductive of cerebro-spinal meningitis.

*Dr. G. G. Crawford* reported a case of chronic catarrh of the uterus, successfully treated by the injections of permanganate of potash in solution. My patient, the mother of two children, had been subjected to various modes of treatment, by other physicians who seem to have mistaken her disease for ordinary leucorrhœa. Vaginal washes, nitrate of silver to the os, and a tannin tampon had been used without benefit. This is but one of several cases, recently coming under my notice, where catarrh of the womb was mistaken for common leucorrhœa, and where vaginal applications rather aggravated than healed the complaint. The instrument used by me was an ordinary uterine syringe, with which the liquid was gently thrown into the cavity of the womb, allowed to remain a few moments, and then re-drawn into the instrument. Having used both the nitrate of silver and permanganate of potash, I feel no hesitation in awarding the palm to the latter remedy, especially when the uterine discharge is of a fetid character. In this class of cases, its effects are peculiarly happy. In the case here reported, iron and Scotch ale were the only general remedies employed. The injections were repeated twice a week for two months when the patient was discharged, in perfect health, after having suffered from the disease for upwards of two years.

---

Condy's Fluid as a Dressing to Wounds.—Condy's Fluid is quite as good as Carbolic Acid as a dressing to wounds, and is much more pleasant.

